# Relationship of immune-inflammatory pathway and stress-induced depression with neural regeneration

**DOI:** 10.4103/NRR.NRR-D-25-00324

**Published:** 2025-11-25

**Authors:** Qingying Yu, Zhaoyu Chen, Jing Zhang, Yu Cao, Mengjia Sun, Shuyi Ye, Yike Huang, Junchi He, Liping Wang, Kejian Li, Simeng Gu, Peng Sun, Qingjun Zhu, Fushun Wang, Ning Weng, Jason H. Huang

**Affiliations:** 1 Innovation Research Institute of Chinese Medicine, Shandong University of Traditional Chinese Medicine, Jinan, Shandong Province, China; 2 School of Pharmaceutical Sciences, Guangzhou University of Chinese Medicine, Guangzhou, Guangdong Province, China; 3 Department of Chinese Medicine, Shandong Mental Health Center, Shandong University, Jinan, Shandong Province, China; 4 Department of Psychiatry, Shandong Mental Health Center, Shandong University, Jinan, Shandong Province, China; 5 Department of Psychiatry, Upstate Medical University, Syracuse, NY, USA; 6 High-Level Key Disciplines of Traditional Chinese Medicine, National Administration of Traditional Chinese Medicine, Shandong University of Traditional Chinese Medicine, Jinan, Shandong Province, China; 7 Department of Medical Psychology, Jiangsu University Medical School, Zhenjiang, Jiangsu Province, China; 8 Department of Psychology, Sichuan Normal University, Chengdu, Sichuan Province, China; 9 Department of Neurosurgery, Baylor Scott & White Health Center, Temple, TX, USA; 10 Department of Neurosurgery, Baylor College of Medicine, Temple, TX, USA

**Keywords:** inflammation, kynurenine, major depressive disorder, microglia, neural regeneration, neurogenesis, synaptic plasticity

## Abstract

Major depressive disorder is a complex psychiatric condition characterized by mood dysregulation, cognitive impairment, and somatic symptoms. Recent studies underscore the pivotal roles of neural regeneration and inflammation in its pathophysiology. This narrative review synthesizes emerging evidence linking neuroinflammatory pathways and impaired neurogenesis in major depressive disorder. We explore mechanisms including microglial activation, kynurenine pathway dysregulation, synaptic remodeling, and gut–brain axis alterations. Special emphasis is placed on recent discoveries highlighting molecular cross-talk between immune responses and neural plasticity. By mapping these interactions, we aim to advance understanding of major depressive disorder subtypes and support the development of inflammation-targeted therapies.

## Introduction

Major depressive disorder (MDD) affects over 350 million individuals globally, posing major social and economic burdens (Bai et al., 2024). By 2030, MDD is projected to be the most prevalent disorder globally. Significant progress has been made in understanding its pathology and treatment, with various mechanisms being proposed, including neurotrophic neurogenesis, inflammation, oxidative stress, and gut microbiota dysregulation (Jiang et al., 2022; Fries et al., 2023). Recent treatment advancements include US Food and Drug Administration-approved interventions such as electrical and magnetic stimulation, as well as ketamine. However, the exact pathophysiological mechanisms remain highly debated, making treatment increasingly challenging.

The symptoms of MDD are heterogeneous, characterized by persistent sadness, mood instability, appetite loss, cognitive impairment, low self-esteem, sleep disturbances, weight fluctuations, psychomotor agitation, and suicidal ideation. Traditional hypotheses centered on monoaminergic dysfunction have failed to fully account for clinical heterogeneity or delayed treatment response (Serafini et al., 2023). In recent years, interest has shifted toward neuroimmune mechanisms, including the interaction between inflammation and impaired neural regeneration.

Recent studies have shown that inflammation is a significant factor in antidepressant resistance and a major risk factor for suicide in MDD patients (Sha et al., 2023), leading some researchers to propose that MDD is a “microglial disease.” Given the presence of the blood–brain barrier (BBB), the brain was traditionally considered an immune-privileged organ. However, new findings reveal that peripheral immune cells can cross the BBB and modulate neural activity. Postmortem studies of depressed suicidal patients have identified increased microglia activation, along with elevated inflammatory cytokine expression in key brain regions (Yamamoto et al., 2024). Moreover, clinical and animal studies have demonstrated elevated inflammatory markers such as C-reactive protein, including interleukin (IL)-1β, IL-6, and tumor necrosis factor (TNF)-α in MDD (Palepu et al., 2024; Miura et al., 2025; Zhang et al., 2025), reinforcing the link between inflammation and depression.

Prolonged or excessive inflammatory responses interfere directly or indirectly with the normal process of neurogenesis through mechanisms such as microglial activation and the release of inflammatory mediators (Alonso et al., 2024). Elevated levels of inflammatory cytokines, including IL-1β, IL-6, and TNF-α, can suppress the proliferation and differentiation of adult neural stem cells, reduce the number of newborn neurons, and consequently impair hippocampal structure and function. Additionally, specific knockout of the IL-10 gene in mice led to a 50% reduction in DCX^+^ cell numbers, resulting in depressive-like phenotypes that were not reversed by fluoxetine treatment (Chen et al., 2024). Furthermore, epigenetic modifications may interact with inflammatory pathways by influencing inflammatory responses and immune regulation, collectively modulating neurogenesis (Zhang and Cao, 2021). Gut microbiota and their metabolites (e.g., short-chain fatty acids) can promote hippocampal neurogenesis by affecting neurotransmitter production, modulating the immune system, and crossing the blood-brain barrier (Agirman et al., 2021). These factors intertwine to form a complex regulatory network that influences the process of neurogenesis and its role in psychiatric disorders.

Despite the mounting evidence supporting the role of inflammation in MDD, critical questions remain regarding its relationship with neurogenesis. This review emphasizes the conceptual novelty of integrating these two domains, proposing that inflammatory signaling disrupts neuroplasticity and contributes to antidepressant resistance. Particularly, dysregulated microglial function, altered neurotrophic support, and stress-induced epigenetic changes represent central pathogenic mechanisms. Here, we synthesize evidence supporting this framework and identify knowledge gaps critical for translation.

## Search Strategy

A literature search was conducted using PubMed and Web of Science (Clarivate platform) from January 2015 to April 2025. Search terms included major depressive disorder, neurogenesis, inflammation, microglia, synaptic plasticity, epigenetics, and kynurenine pathway. Only English-language peer-reviewed articles were included. Both preclinical and clinical studies were considered. Reference lists of relevant reviews were also screened. The narrative format was chosen to allow thematic synthesis across multidisciplinary findings.

## Stress-induced Immune-Inflammatory Responses in Major Depressive Disorder: Causes and Consequences

Stress is a key factor in the development of MDD, particularly early-life stress. Numerous studies have shown that prolonged exposure to chronic stress, such as childhood abuse, negative life events, financial strain, and severe physical illness, can significantly increase vulnerability to MDD. Stress-induced neurotransmitter release (e.g., norepinephrine, glutamate, and adenosine triphosphate) activates microglia and astrocytes (Wang et al., 2023), leading to immune system activation and the release of inflammatory cytokines. This immune response is a crucial mechanism in the development of depression.

Supporting this, patients with autoimmune diseases or inflammatory conditions frequently exhibit depressive symptoms. Stress also induces transient morphological and functional changes in microglia, but chronic stress can lead to long-term adverse effects (Gaspar et al., 2022). Acute stress activates the immune response via catecholamines and glucocorticoids, triggering microglial activation in the prefrontal cortex, hippocampus, thalamus, and midbrain periaqueductal gray matter. Chronic stress, on the other hand, leads to microglial activation in regions such as the caudate nucleus, amygdala, hippocampus, nucleus accumbens, and cingulate cortex (Fu et al., 2025; Melkumyan et al., 2025; Mongi-Bragato et al., 2025; Uranova et al., 2025; Wu et al., 2025).

### Stress-induced microglial activation

In the early stages of stress, microglial proliferation and activation occur, but prolonged chronic stress can lead to microglial apoptosis and reduced expression of activation markers. Stress-induced microglial activation is predominantly observed in brain regions implicated in depression, including the prefrontal cortex, amygdala, and hippocampus (Franklin et al., 2018). This activation is associated with elevated levels of pro-inflammatory cytokines (**Table 1**).

**Table 1:** Mechanisms of depressogenic effects of stress on microglia

Modeling methods and model types	Brain regions	Pharmacological mechanisms	Reference
Maternal separation	Prefrontal	IL-1β	Wang et al., 2017
Chronic unpredictable stress	Prefrontal, hippocampus	Nuclear factor-κB, IL-1β, IL-1, IL-6, TNF-α, and NLPR3	Deng et al., 2015; Zhang et al., 2015; Liu et al., 2018; Parul et al., 2021
Chronic social defeat stress	Hippocampus	IL-6, NLRP3	Ito et al., 2017
Chronic unpredictable mild stress	Hippocampus	TLR4, TNF-α, IL-6, IL-1β	Guo et al., 2019
Lipopolysaccharide	Primary microglia, BV2 cell line, midbrain, and hippocampus	Nuclear factor erythroid 2-related factor 2, inducible nitric oxide synthase, IL-6, TNF-α, IL-1β, nuclear factor-κB, NLRP3	Han et al., 2021
Aversive stimuli	Lateral habenula	Allograft inflammatory factor 1	Fu et al., 2021
Chronic restraint stress	Hippocampus	Allograft inflammatory factor 1	Yi et al., 2020

The exact signaling mechanisms underlying stress-induced microglial activation remain under investigation. However, pattern recognition receptors, ion channels, purinergic receptors, CX3C chemokine receptor 1, and adrenergic receptors have been identified as key pathways. Stressors can trigger pathogen-associated molecular patterns (e.g., lipopolysaccharide) and damage-associated molecular patterns (e.g., S100 proteins, heat shock proteins, and adenosine triphosphate). Microglia, equipped with pattern recognition receptors, recognize these danger signals and amplify neuroinflammation. IL: Interleukin; NLPR3: NOD-like receptor thermal protein domain associated protein 3; TLR4: Toll-like receptor 4; TNF-α: tumor necrosis factor alpha.

Studies have found that stress significantly upregulates hippocampal high-mobility group protein B1 (HMGB1) and NLRP3 inflammasome expression, leading to microglial activation (Kaufmann et al., 2017; Wang et al., 2020a). Conversely, HMGB1 antagonists or Toll-like receptor 4 (TLR4) blockade prevent stress-induced neuroinflammation (Weber et al., 2015; Frank et al., 2016; Franklin et al., 2018; Nie et al., 2018). Chronic unpredictable mild stress (CUMS) induces depression-like behavior through activation of pathways such as phosphatidylinositol 3-kinase (PI3K)/protein kinase B, glycogen synthase kinase 3, and TLR4, with TLR4 knockdown reducing cytokine levels in hippocampal tissues (Cheng et al., 2016).

Microglia activation-mediated neuroinflammation suppresses adult hippocampal neurogenesis (Zhang et al., 2020a). Disrupting microglial function can induce a cascade effect in the inflammatory system and inhibit adult hippocampal neurogenesis, thereby increasing susceptibility to stress and depression (Anacker et al., 2018; Zhang et al., 2021). Studies have shown that microglia can regulate neurogenesis by secreting various factors that modulate the neurogenic microenvironment (Li et al., 2025; Zhao et al., 2025). The activation of hippocampal microglia perpetuates neuroinflammation and the release of neurotoxic inflammatory mediators, thereby impairing hippocampal neurogenesis (Zhang et al., 2020a). Indeed, after stress exposure, the concentrations of IL-6, IL-1β, and TNF-α in the hippocampus of CUMS mice were higher than those in Ctrl mice (He et al., 2024). By releasing these cytokines into the neurogenic microenvironment, microglia not only suppress the proliferation and survival of neural stem cells but also hinder their differentiation into neurons (Liu et al., 2022). All these factors may contribute to alterations in the continuous production of adult-born neurons in the hippocampal DG (Toda et al., 2019).

#### Role of microglia in remyelination

Oligodendrocytes ensheath neuronal axons and form insulating myelin sheaths, which function to provide insulation for rapid electrical impulse conduction along nerve fibers and supply neurotrophic metabolic factors for neuronal health. Oligodendrocyte precursor cells (OPCs) persist throughout life in all brain regions and are the only cell type in the central nervous system capable of differentiating and replacing oligodendrocytes (Hutchinson and Isaacson, 2019). In neurological disorders, oligodendrocytes swell, and the majority undergo cell death (Zuo et al., 2019). Numerous factors can regulate remyelination, including cytokines, growth factors, and neuronal activity (Franklin and Ffrench-Constant, 2017).

Microglia are involved in oligodendrocyte regeneration. Microglia enhance remyelination by directly modulating OPC behavior. After demyelination, microglia rely on the CX3CR1 and RXR-γ receptors to phagocytose myelin debris, promoting the recruitment and differentiation of OPCs at the injury site (Lampron et al., 2015; Natrajan et al., 2015). Additionally, microglia and monocyte-derived macrophages facilitate the remyelination process by creating an environment that supports OPC recruitment and their subsequent differentiation into mature myelin-producing oligodendrocytes (Lloyd and Miron, 2019). Different microglial phenotypes dominate specific stages of focal remyelination. An *in vitro* study has shown that microglia polarize into the M1 phenotype under interferon-γ and lipopolysaccharide (LPS) induction, and IL-1β secreted by M1 microglia promotes OPC differentiation into oligodendrocytes, thereby enhancing remyelination (Lampron et al., 2015). Both M1 and M2 microglia are essential for remyelination and play beneficial roles in the process.

#### Role of microglia in neuronal regeneration

A previous study has shown that after stress, microglia polarize into different phenotypes, clearing cellular debris through phagocytosis, synthesizing and releasing neurotrophic factors to promote neuronal activity, and even inducing the migration of neural precursor cells from other brain regions. These processes facilitate their proliferation and differentiation into newborn neurons, thereby protecting the nervous system and promoting the recovery of neural function (Jayaraj et al., 2019).

Activated microglia, along with reactive astrocytes and the extracellular matrix, can form glial scars (Moeendarbary et al., 2017). M2-type microglia can induce the secretion of FIZZ1 protein and heparin-binding lectin protein, preventing the degradation of glial scars (Arcuri et al., 2017). Neural circuits are the foundation of information transmission between brain neurons. Stress disrupts neural circuits, and microglia play a crucial role in their reconstruction. M2-type microglia promote neural circuit remodeling, facilitating the recovery of neurological function (Sandvig et al., 2018). Toll-like receptors (TLRs) on the surface of microglia are pattern recognition receptors with modifiable activity (Liu et al., 2019). Microglia recognize pro-inflammatory factors via surface TLR4 and activate the nuclear factor kappa-B (NF-κB) pathway to participate in the release of inflammatory mediators. However, treatment with indigo can inhibit the TLR4/NF-κB signaling pathway, promoting the shift of microglia from the M1 to the M2 phenotype, thereby mitigating neural damage and exerting neuroprotective effects (Ye et al., 2019). Therefore, timely intervention to modulate microglial polarization post-stress can harness their neuroprotective potential (Kanazawa et al., 2017).

IL-4 is crucial for microglial polarization and neuronal recovery, promoting the shift of microglia toward the M2 phenotype and enhancing their phagocytic clearance of damaged cells, while also inducing the regeneration of injured neural tissue (Zhao et al., 2015; Liu et al., 2016). Activated microglia can express numerous neurotrophic factors that facilitate neuronal regeneration (Zhou et al., 2020), including calmodulin, osteopontin, platelet-derived growth factor, epidermal growth factor, fibroblast growth factor-2, ciliary neurotrophic factor, activin-A, glia-derived growth factor, endothelin-2, insulin-like growth factor 1, neurotrophins, brain-derived neurotrophic factor, and neurotrophin-3, all of which contribute to post-injury neuronal recovery (Sousa-Victor et al., 2018). Certain neurotrophic factors, such as nerve growth factor, can immunomodulate microglia — for example, nerve growth factor regulates microglial surface molecules, particularly by downregulating major histocompatibility complex class II levels, thereby reducing neuroinflammation and promoting neuronal regeneration (Giera et al., 2018).

**Figure 1:**
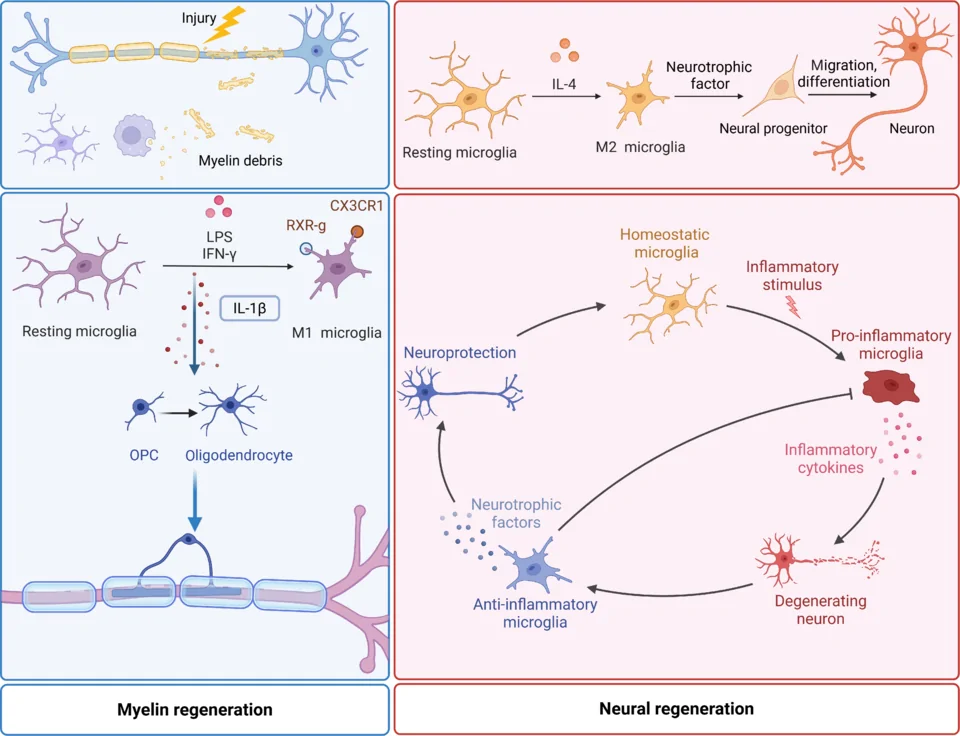
Neurogenic role of microglia. Demyelination occurs following damage to myelin sheaths caused by injury or inflammation. During remyelination, OPCs are recruited, proliferate, and differentiate into mature oligodendrocytes, which subsequently form new myelin sheaths. Microglia become activated in response to stress and release factors that impair oligodendrocyte maturation, thereby reducing myelination. Furthermore, microglia-derived neurotoxic factors exacerbate neurotoxicity and promote neuronal death. Created with BioRender.com. CX3CR1: CX3C chemokine receptor1; IFN-γ: interferon-γ; IL-1β: interleukin-1β; IL-4: interleukin-4; LPS: lipopolysaccharide; OPC: oligodendrocyte precursor cell; RXR-g: retinoid X receptor gamma.

In the adult central nervous system (CNS), neural stem cells are primarily located in two regions: the subventricular zone and the subgranular zone of the hippocampal dentate gyrus (Delgado et al., 2016). Upon CNS injury, self-renewing and multipotent neural precursor cells proliferate and migrate from other CNS regions to the injury site, differentiating into new neurons to replace damaged neural cells. Studies have shown that in neuroinflammation, M1-type microglia negatively regulate the activity of neural precursor cells and downregulate cytokine levels associated with their differentiation, whereas M2-type microglia promote the migration of neural precursor cells and their differentiation into neurons, facilitating neuronal regeneration. Experimental evidence demonstrates that indomethacin can modulate microglial phenotypes, promoting the migration and neuronal differentiation of neural precursor cells in the subventricular zone. Neural stem cells can also influence microglial function by driving their migration toward the subventricular zone and regulating their proliferation and activation through the release of vascular endothelial growth factor, though the underlying mechanisms require further investigation. The bidirectional regulatory interaction between microglia and neural stem cells may represent a novel therapeutic target for promoting neurogenesis (**Figure 1**).

Pharmacological interventions targeting inflammation have shown promise. Anti-inflammatory agents such as geraniol and sinomenine significantly reduce pro-inflammatory cytokine levels and alleviate depression-like behavior in mice (Deng et al., 2015; Liu et al., 2018). Additionally, antidepressants such as fluoxetine have been found to decrease TNF-α, IL-6, and IL-1β mRNA levels in LPS-induced depression models (Duda et al., 2017; Dionisie et al., 2021). These findings highlight the crucial role of immune-inflammatory responses in stress-induced depression and suggest that targeting inflammatory pathways may offer potential therapeutic strategies.

### Glymphatic system

The glymphatic system, a recently discovered cerebrospinal fluid-interstitial fluid exchange network (Holstein-Rønsbo et al., 2023), plays a vital role in clearing large soluble proteins and maintaining brain homeostasis (Iliff et al., 2015). Functionally similar to the peripheral lymphatic system, it relies on glial cells, particularly astrocytes, for its operation (Jiang et al., 2022).

Recent research has linked glymphatic system dysfunction to neuroinflammation and depression (Iliff et al., 2015). The system facilitates the removal of pro-inflammatory cytokines, such as TNF-α and IL-1β (Hsu et al., 2021). Impairments in glymphatic function result in the accumulation of reactive oxygen species (ROS) and pro-inflammatory signals, leading to cellular damage and apoptosis in major depression (Roomruangwong et al., 2017).

Aquaporin-4 polarization is essential for glymphatic function (Rasmussen et al., 2018). Aquaporin-4 facilitates physiological water transport and is involved in inflammatory signaling. Chronic stress has been shown to induce glymphatic pathway dysfunction, potentially bridging the gap between inflammation and monoamine dysregulation in MDD (Roomruangwong et al., 2018; Harrison et al., 2020). CUMS-induced norepinephrine release disrupts aquaporin-4 polarization, inhibits glymphatic clearance, and promotes oxidative stress, inflammation, and depression-like symptoms.

**Figure 2:**
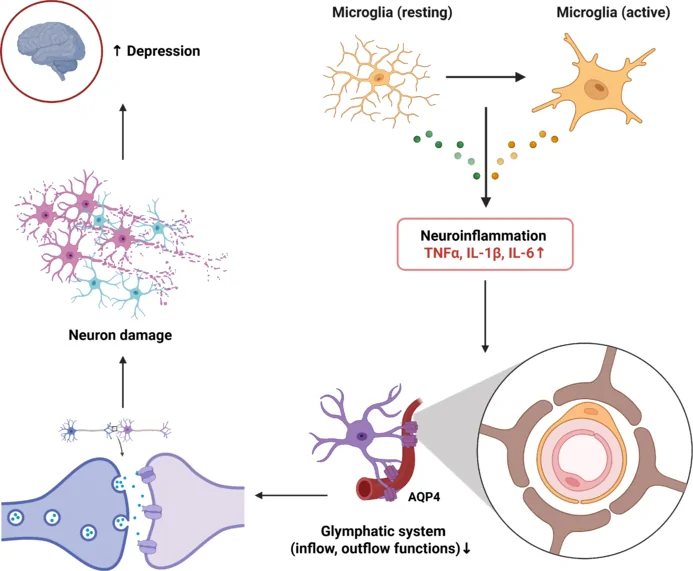
Stress leads to abnormal functioning of the glial lymphatic system. Stress leads to sustained activation of microglia in the central nervous system. Activated microglia secrete a variety of pro-inflammatory factors, including TNF-α, IL-1β, and IL-6, which activate and affect the clearance function of the glymphatic system. The water channel protein AQP4 on astrocytes regulates astrocyte-microglia contact in neuroinflammation and is a key contributor to the clearing efficiency and homeostasis of the glymphatic system. Dysfunction of the glymphatic system leads to disturbances in neurotransmitter release, which in turn causes necrosis of glial cells and neurons, inducing mental and behavioral disorders. Created with BioRender.com. AQP4: Aquaporin-4; IL-1β: interleukin-1β; IL-6: interleukin-6; TNF-α: tumor necrosis factor-α.

Norepinephrine-induced overactivation of microglia and astrocytes further impairs glymphatic function (Saller et al., 2012; Chen et al., 2018), leading to increased TNF-α, IL-1β, and IL-6 levels (Zhan et al., 2024; **Figure 2**). Recent studies suggest that norepinephrine significantly disrupts glymphatic function, resulting in neuronal necrosis (Louveau et al., 2015; Rasmussen et al., 2018). However, further research is needed to elucidate how chronic stress contributes to depression via glymphatic dysfunction.

## Genetics and Epigenetics

Adverse childhood experiences are strongly associated with increased depression susceptibility. While stress plays a crucial role in affective disorders, its precise mechanisms remain unclear. Recent epigenetic studies suggest that early-life adversity induces long-term mental and physical health effects by altering gene expression (Wang et al., 2021a).

Epigenetic modifications regulate the response of genome to environmental stressors. A previous study has found that early-life stress induces DNA methylation changes in neuromodulator receptor genes, such as those encoding adrenocorticotropic hormone receptors and monoamine oxidase (Xu et al., 2020). These modifications impact neurotransmitter and hormone receptor function, potentially contributing to affective disorders.

The hypothalamic–Pituitary–Adrenal (HPA) axis, a central regulator of stress responses, is also influenced by epigenetic changes. Alterations in HPA function due to epigenetic modifications have been observed in both humans and animal models. These changes lead to dysregulated monoamine neurotransmission and neuroendocrine dysfunction (Kronman et al., 2021; Padilla et al., 2023), key contributors to MDD pathophysiology.

### DNA methylation

DNA methylation of immune-related genes has been strongly linked to depression. Genome-wide association studies have identified variants in immune genes, such as P2X purinoceptor 7 (Wingo et al., 2021), that may influence depression susceptibility. Functional single nucleotide polymorphisms in the IL-6 receptor promoter region correlate with depression severity and inflammatory marker levels (IL-6 and C-reactive protein) (Khandaker et al., 2018).

Epigenome-wide association studies have demonstrated that early-life stress is associated with DNA methylation of inflammation-related genes (Kuan et al., 2017). For example, IL17RA methylation (a receptor for IL-17A produced by T lymphocytes) correlates with childhood adversity in depressed patients (Ramaswami et al., 2020). Similarly, methylation changes in Ubiquitin thioesterase OTU1 (YOD1), a gene involved in inflammatory regulation, have been identified in postmortem brains of patients with late-onset depression (Wong et al., 2019; Hüls et al., 2020).

Methylation of cytokine-encoding genes, including IL-6, granulocyte-macrophage colony-stimulating factor 2, IL-8, and IL-4, has been linked to early-life stress (Janusek et al., 2017; Zou et al., 2020). These findings provide strong evidence that immune-inflammatory responses play a central role in depression pathogenesis.

Moreover, prospective analyses of genome-wide gene expression have identified immune-related genes, such as Janus kinase 2 and chimerin 2, as differentially expressed in selective serotonin reuptake inhibitor treatment responders versus non-responders. These genes regulate intrinsic and adaptive immunity and may impact hippocampal neurogenesis (Ju et al., 2019), further supporting the immune-inflammation hypothesis in depression.

### Histone modifications

Recent studies suggest that histone modifications play a crucial role in regulating microglial plasticity and polarization. One key mechanism involves the activation of bromodomain and extraterminal domain proteins, which regulate various brain genes, including those involved in neurotrophic signaling, cytokine production, ion channel activity, synaptic receptor function, and immediate-early gene responses (Singh and Sartor, 2020). These processes are essential for neurodevelopment, neuroinflammation, myelination, learning, and memory, and are closely linked to neurodegenerative diseases, psychiatric disorders, and central nervous system tumors.

Bromodomain and extraterminal inhibitors have been shown to modulate inflammatory signaling pathways, particularly by suppressing NF-κB activation. Bromodomain-containing protein 4 (BRD4) acts as a co-activator of NF-κB transcription by binding specifically to acetylated RelA (Wibisana et al., 2022). Chromatin immunoprecipitation experiments have demonstrated that BRD4 directly associates with promoter regions of inflammatory cytokine genes across various cell types, from fibroblasts to macrophages. Notably, bromodomain and extraterminal inhibitors reduce BRD4 binding, leading to decreased pro-inflammatory cytokine expression (Zhong et al., 2025).

The BRD4 inhibitor JQ1 has been shown to suppress glial activation (Zhou et al., 2019) and attenuate LPS-induced pro-inflammatory cytokine elevation by modulating inflammatory signaling pathways (Wang et al., 2018). These findings suggest a potential role for BRD4 in stress-induced neuroinflammation and highlight its influence on depression-like behavior through increased neuroinflammation in the prefrontal cortex and hippocampus (Wang et al., 2021b).

In addition to BRD4, histone-lysine N-methyltransferase EZH2 (Enhancer of Zeste Homolog 2) is another key epigenetic regulator of neuroinflammation. EZH2 inhibition reduces pro-inflammatory mediator expression (Arifuzzaman et al., 2017; Zhang et al., 2018). Tazemetostat, a selective EZH2 inhibitor, competitively binds to S-adenosylmethionine, preventing H3K27me3 trimethylation and downregulating critical inflammatory mediators following microglial stimulation by LPS (Arifuzzaman et al., 2017).

Moreover, EZH2 directly suppresses suppressor of cytokine signaling 3 (SOCS3) expression, thereby influencing NF-κB activation through TLR-related pathways. Animal studies suggest that EZH2-mediated H3K27 trimethylation is involved in depressive-like behaviors induced by chronic stress by modulating microglial activation and cytokine expression (Wang et al., 2020b). EZH2 also binds to GAS5, mediating the inhibition of NRF2 and SOCS2, further contributing to depressive pathology (Li et al., 2022).

### MicroRNA and other non-coding RNA

MicroRNAs (miRs) play a pivotal role in neuroinflammation and depression, mediating interactions between immune cells and stress-related signaling pathways. Overexpression of miR-96 in the hippocampal CA1 region promotes oxidative stress and neuroinflammatory responses, characterized by increased IL-1β, TNF-α, and malondialdehyde production (Sun et al., 2020).

miR-155 regulates inflammatory pathways mediated by interferon-alpha/beta receptors, TNFR, IL-1R, and TLRs. It degrades anti-inflammatory factors, disrupting serotonin (5-HT) metabolism and amplifying inflammatory cytokine production. Additionally, miR-155 promotes inflammation by degrading SOCS1 mRNA, leading to downregulation of SOCS1 protein and the loss of its inhibitory effect on inflammatory signaling.

Brás et al. (2022) found that miR-342 expression was upregulated in the hippocampus of stressed adult male rats. Its expression level positively correlated with passive coping responses, microglial activation, and TNF-α expression, suggesting that miR-342 may drive TNF-α-mediated microglial activation and could serve as a potential therapeutic target for inflammation-associated depression.

Other miRs involved in inflammatory signaling include: miR-135a-5p, which attenuates inflammation and apoptosis via the stromal cell-derived factor 1 signaling pathway, exhibiting protective effects against neuroinflammation (Guo et al., 2020); miR-146a, which regulates allograft inflammatory factor 1, a gene elevated in depression. miR-146a modulates inflammatory responses by downregulating IL receptor-associated kinase, TNF-α, IL-1β, and inducible nitric oxide synthase. In animal models, miR-146a inhibition of microglial activation improved depressive-like behaviors (Liu et al., 2021). miR-124, which directly targets STAT3 to inhibit inducible nitric oxide synthase expression in microglial cells. Upregulation of miR-124 in the hippocampus has been shown to regulate microglial activity, reducing inflammation and improving depressive-like behaviors in CUMS mice (Lou et al., 2019). miR-135a, which mitigates depressive symptoms by inhibiting TLR4 expression in the hippocampus (Ding et al., 2021).

Collectively, these findings suggest that miRs post-transcriptionally regulate immune and inflammatory responses in depression, making them promising targets for novel antidepressant strategies.

## Monoamine Theory and the Kynurenine Pathway

The monoamine hypothesis, first proposed in the 1950s, posits that deficiencies in monoamines — norepinephrine, dopamine, and 5-HT — contribute to affective disorders. Despite the identification of additional mechanisms, first-line antidepressants still primarily target monoamine neurotransmission.

Building on our prior research (Gu et al., 2019), the monoamine hypothesis requires refinement to better capture the distinct emotional profiles associated with each neurotransmitter. Dopamine emerges as the chemical correlate of joy and motivation, while norepinephrine orchestrates primal “fight-or-flight” responses, intimately tied to fear and anger. In contrast, 5-HT modulates a calmer spectrum of experiences — sedation, sleep, and emotional equilibrium (Han et al., 2023).

However, third-generation antidepressants, such as selective serotonin reuptake inhibitors, selectively enhance serotonergic activity by blocking 5-HT reuptake. The role of serotonin in depression remains controversial, given the diversity of its 14 receptor subtypes.

### Serotonin and the kynurenine pathway

Emerging evidence suggests that serotonin neurotransmission is modulated by immune mediators in MDD. Pro-inflammatory cytokines stimulate indoleamine 2,3-dioxygenase in astrocytes, diverting tryptophan metabolism toward kynurenine production rather than serotonin synthesis (Liu et al., 2015). Similarly, high peripheral levels of glucocorticoids activate tryptophan 2,3-dioxygenase (Stone and Williams, 2024), further depleting tryptophan availability for serotonin biosynthesis (Maguire, 2019).

This shift in metabolism contributes to inflammation-induced depression by reducing serotonin levels (Medina-Rodriguez et al., 2018). Zhang et al. (2020b) demonstrated that chronic stress significantly increased hippocampal kynurenine levels, which were accompanied by astrocyte NLRP2 inflammasome activation. Notably, NLRP2 knockdown mitigated kynurenine-induced depressive-like behaviors in mice.

Inflammatory mediators such as TNF-α and IL-1β further exacerbate serotonin depletion by activating p38 mitogen-activated protein kinase (MAPK), which enhances 5-HT transporter function, increasing serotonin reuptake and reducing synaptic 5-HT levels. This mechanism provides a direct link between inflammation and serotonin dysregulation in depression.

### Kynurenine pathway and glutamate dysregulation

Pro-inflammatory cytokines also impact the glutamatergic system via the kynurenine pathway. Indoleamine 2,3-dioxygenase catalyzes tryptophan metabolism into kynurenine, which is subsequently converted into kynurenic acid and quinolinic acid within the central nervous system. Kynurenic acid has neuroprotective properties, acting as an N-methyl-D-aspartate receptor antagonist. Quinolinic acid, in contrast, is neurotoxic. It directly activates N-methyl-D-aspartate receptors, increases glutamate release, inhibits glutamate uptake by astrocytes, and promotes excitotoxicity.

**Figure 3:**
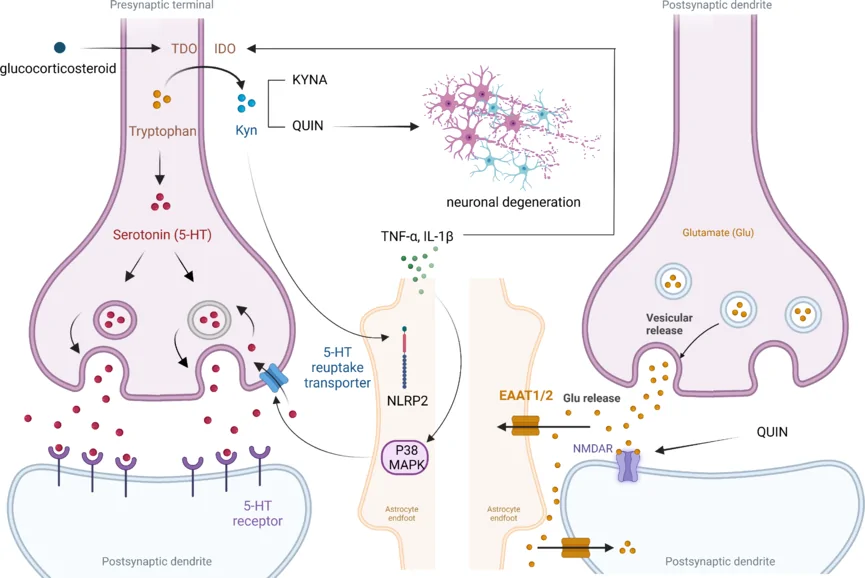
Relationship between monoamine and canine uridine pathways and inflammation. Activating the inflammatory response, which involves astrocytes as well as the glymphatic system, results in the secretion of inflammatory cytokines such as TNF-α and IL-1β. These inflammatory proteins and associated signaling pathways, which include P38 MAPK cause reverse 5-HT efflux through activate 5-HT transporter expression and function. Astrocytes also produce increased levels of pro-inflammatory cytokines, which stimulate the metabolism of tryptophan to Kyn, thereby driving the production of quinolinic acid, which activates NMDAR to stimulate the release for Glu and inhibits Glu uptake from glial cells via ETTA, further increasing Glu accumulation in the cytosol. Glu spills over into the extrasynaptic space to bind to the NMDAR, ultimately leading to neuronal degeneration and depression. Created with BioRender.com. 5-HT: Serotonin; EAAT1/2: excitatory amino acid transporter 1/2; Glu: glutamate; IDO: indoleamine 2,3-dioxygenase; IL-1β: interleukin-1 beta; Kyn: kynurenine; KYNA: kynurenic acid; MAPK: mitogen-activated protein kinase; NLRP2: NACHT-, LRR- and PYD-containing protein 2; NMDAR: N-methyl-D-aspartate receptor; QUIN: quinolinic acid; P38: p38 protein kinase; TDO: tryptophan 2,3-dioxygenase; TNF-α: tumor necrosis factor-α.

This excess glutamate activity results in oxidative stress, inflammatory signaling, and neuronal degeneration, all of which contribute to depressive symptoms (Oh et al., 2023; Lee et al., 2025; Xiao et al., 2025; **Figure 3**). Notably, quinolinic acid levels correlate with IL-6 concentrations in suicidal patients, and cerebrospinal fluid analyses reveal a threefold increase in quinolinic acid levels in suicide attempters compared to controls (Brundin et al., 2017).

IL-6 receptors are widely expressed on serotonergic neurons in the hippocampus and other brain regions, further supporting the role of inflammation in serotonin and kynurenine pathway dysregulation. Rapid IL-6 administration has been shown to increase 5-HT release in the rat striatum (Brundin et al., 2017), suggesting a possible mechanism by which inflammation exacerbates depression and suicidality.

## Hypothalamic–Pituitary–Adrenal Axis

### Hypothalamic–pituitary–adrenal axis dysregulation and inflammatory responses

Dysfunction of the HPA axis plays a central role in depression pathogenesis (Zerroug et al., 2025). Studies indicate that 20% to 80% of depressed patients exhibit HPA axis abnormalities, including pituitary and adrenal gland enlargement and hypercortisolism. Clinical assessments often reveal increased cortisol levels, abnormal dexamethasone suppression test results, and downregulated glucocorticoid receptor (GR) function — all hallmarks of HPA axis hyperactivity (Appleton, 2025; Su et al., 2025; Zerroug et al., 2025).

Cerebrospinal fluid analyses of depressed individuals show elevated corticotropin-releasing hormone (CRH) levels, suggesting that increased CRH secretion underlies HPA axis dysfunction in depression. Emerging research highlights neuro-endocrine-immune dysregulation as a key contributor to depressive disorders. Early-life stress has lasting effects on inflammatory responses and neuroendocrine expression patterns (Baumeister et al., 2016), leading to persistent HPA axis alterations.

When an individual experiences trauma, the HPA axis is activated, stimulating the release of glucocorticoids (GCs) from the adrenal glands. Prolonged GC elevation can activate microglia, leading to the release of GRs and mineralocorticoid receptors from microglial cells. GR plays a dual role: it regulates HPA axis feedback and modulates inflammatory responses (Cattaneo and Riva, 2016). However, GR dysfunction leads to excessive GC production, exacerbating inflammation.

Elevated GC levels have been linked to increased LPS-induced NF-κB activation and higher expression of pro-inflammatory cytokines (TNF-α, IL-1β, and inducible nitric oxide synthase) in the hippocampus and frontal cortex. These changes contribute to neuroinflammation, inhibition of neurotrophic factor production, neuronal apoptosis, and hippocampal dysfunction (Sorrells et al., 2014), which are all associated with depressive-like behaviors.

In line with this, Kim et al. (2016) found that chronically elevated GCs led to increased NF-κB, pro-inflammatory cytokines, and MAPK activation, ultimately sustaining chronic inflammation. This suggests that neuroimmune-endocrine interactions contribute to depression, although further research is needed to fully elucidate the reciprocal relationship between inflammation and HPA axis dysregulation.

### Inflammation-induced hypothalamic–pituitary–adrenal axis dysregulation

Inflammation itself can exacerbate GR dysfunction, leading to persistent GR resistance into adulthood (Baumeister et al., 2016). Under the influence of chronic stress and immune activation, the release of peripheral inflammatory cytokines significantly affects HPA axis function by altering the immune-neuroendocrine network.

**Figure 4:**
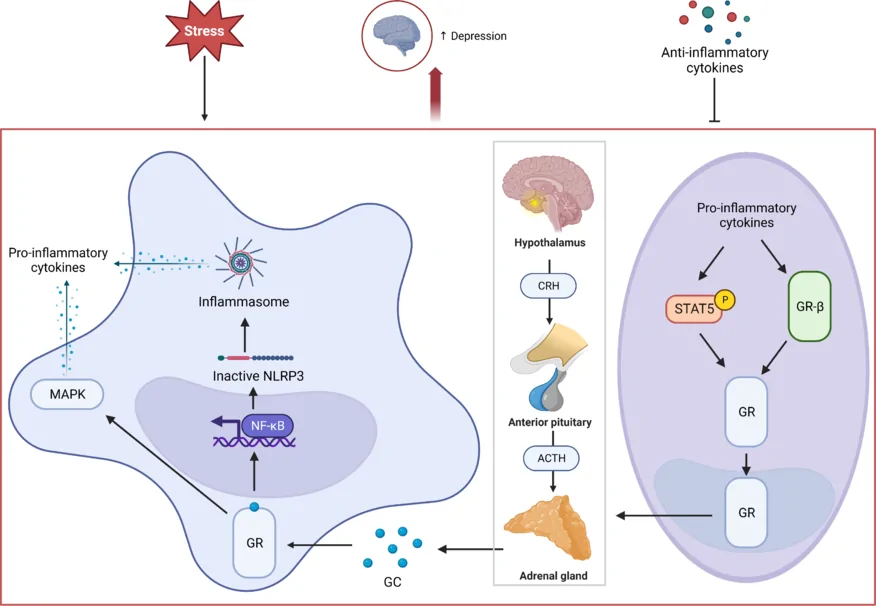
Relationship between the HPA axis and inflammation. In all, the HPA axis interacting with the immune system to form a sustained inflammatory response might be the reason for stress induced depression. Stress exposure can activate the immune system to induce inflammatory responses such as increased release of peripheral inflammatory factors, and lead to impaired negative feedback regulation of the HPA axis and thus depression. Created with BioRender.com. ACTH: Adrenocorticotropic hormone; CRH: corticotropin releasing hormone; GC: glucocorticoid; GR: glucocorticoid receptor; GR-β: glucocorticoid receptor-β; HPA: hypothalamic-pituitary-adrenal; MAPK: mitogen-activated protein kinase; NF-κB: nuclear factor kappa-B; NLRP3: NACHT-, LRR- and PYD-containing protein 3; P: phosphorylation; STAT5: signal transducer and activator of transcription 5A.

Pro-inflammatory cytokines impede GR function via STAT5 phosphorylation, and activation of GRβ, an inactive form of the receptor, further disrupts GR signaling (Kim et al., 2016). Consequently, impaired GR function leads to loss of HPA axis negative feedback regulation, perpetuating sustained GC secretion and hippocampal GR downregulation (**Figure 4**).

This chronic overactivation of the HPA axis fosters a vicious cycle of neuroendocrine dysfunction and neuroinflammation. The “neuroendocrine-immune network” theory posits that immune system dysregulation and inflammation influence the neuroendocrine system, disrupting HPA metabolism and contributing to depressive symptomatology (Yano et al., 2015).

Supporting this, studies have identified strong correlations between depression and elevated pro-inflammatory cytokines (IL-1β, IL-2, and IL-6). IL-1β, in particular, stimulates CRH secretion, suggesting that microglia-derived IL-1β may serve as an HPA axis activator. Depressed patients with chronic stress exposure often exhibit HPA axis hyperactivity, accompanied by increased CRH and elevated plasma cortisol levels (Menke, 2019), which further exacerbate depressive symptoms.

During immune activation, anti-inflammatory cytokines help counteract the effects of pro-inflammatory cytokines. These regulatory mechanisms reach the brain via the BBB, potentially reducing neuronal damage and improving depressive symptoms.

Interestingly, IL-1 receptor antagonist (IL-1RA), a selective inhibitor of IL-1 signaling, has been found to correlate positively with IL-1 levels in depressed patients, suggesting an immune-activated state. IL-1RA has been detected in the hypothalamus and other neuroendocrine-regulating brain regions (de Vries et al., 2016), reinforcing the hypothesis that central IL-1RA plays a role in modulating stress responses.

Further evidence for the immune-endocrine interaction in depression comes from studies showing that the immune system is activated before HPA axis stimulation following stress exposure. Kim et al. (2016) observed that immune activation directly triggers HPA axis activity, reinforcing the idea that pro-inflammatory and anti-inflammatory cytokines are major contributors to immune-mediated depression.

### Bidirectional relationship between inflammation and hypothalamic–pituitary–adrenal axis dysfunction

A key question remains: Does stress-induced inflammation impair GR function, leading to a subsequent inflammatory response? Or does an inflammatory response first impair GR function, perpetuating a feedback loop?

The bidirectional interplay between inflammation and HPA axis dysfunction is well-documented. Meta-analyses suggest that early-life stress alters GR function, leading to persistent inflammation (Baumeister et al., 2016). Chronic stress contributes to prolonged pro-inflammatory cytokine release, further reinforcing GR resistance and HPA axis overactivity.

At the same time, GR dysfunction exacerbates inflammation, leading to prolonged secretion of inflammatory cytokines (TNF-α, IL-6, and IL-1β). This creates a self-perpetuating inflammatory cycle, contributing to chronic neuroinflammation and HPA axis hyperactivation — hallmarks of stress-induced depression.

Overall, GR functions as a key mediator between stress and depression, perpetuating a cycle of HPA axis dysregulation and inflammatory responses. This chronic feedback loop sustains depressive pathology and represents a potential target for therapeutic intervention.

## Synaptic Transmission and Depression

Recent research highlights synaptic developmental abnormalities as a key contributor to depression pathogenesis (Vose and Stanton, 2017; Li et al., 2020). Disruptions in synaptic plasticity can lead to neuronal atrophy and reduced connectivity, particularly in the hippocampus and prefrontal cortex — two brain regions heavily implicated in depression (Uchida et al., 2018).

Both glutamatergic and GABAergic synaptic transmission are dysregulated in depression, leading to weakened synaptic function and neurodegeneration. Given the role of synaptic plasticity in learning, memory, and mood regulation, many recent studies have incorporated synaptic dysfunction into the evolving framework of antidepressant mechanisms.

### Role of microglia in synaptic plasticity

Microglia, resident immune cells of the brain, play an integral role in synapse formation, pruning, and repair. Under normal conditions, microglia remain in a resting state, yet they continuously monitor synaptic activity and engage in phagocytosis of intersynaptic material to maintain neural homeostasis (Umpierre and Wu, 2021). However, dysfunctional microglia can impair synaptic plasticity, leading to depression-like behaviors.

During acute stress, microglia become activated (Smiley et al., 2025), but in chronic stress, prolonged activation shifts microglia toward apoptosis or dysregulated inflammatory states. Morphological and functional abnormalities in microglia result in the release of pro-inflammatory cytokines, which in turn affect synaptic integrity and neuroplasticity (Riazi et al., 2015). This process is a key contributor to the development of depressive symptoms.

Similarly, astrocytes, which envelop synapses, modulate intersynaptic signaling in response to neurotransmitters such as glutamate and regulatory factors (Flanagan et al., 2021). Depression-like phenotypes are often accompanied by astrocytic dysfunction, including downregulation of synaptophysin-2 and inhibition of synaptic vesicle release, leading to compromised synaptic communication.

### Inflammation and synaptic dysfunction

Pro-inflammatory cytokines play a significant role in disrupting synaptic plasticity, with TNF-α, IL-1β, TNFR1, and cannabinoid receptor type 1 being key mediators in synaptic impairment (Ding et al., 2025). These inflammatory molecules can reduce synaptic connectivity, alter neurotransmitter receptor function, and enhance excitotoxicity via N-methyl-D-aspartate receptor overactivation.

Chronic stress and neuroinflammation are known to elevate pro-inflammatory cytokine expression, which adversely affects synaptic function through pathways such as p38 MAPK and NF-κB signaling (Duman et al., 2016). While low levels of pro-inflammatory cytokines may activate the PI3K/Akt pathway to maintain synaptic plasticity, abnormally high levels activate p38 and NF-κB, leading to synaptic damage and dysfunction (Brüning et al., 2015).

Thus, while inflammatory responses are necessary for normal brain function, their dysregulation can promote depressive pathology by interfering with neurotransmission and synaptic remodeling.

### Potential therapeutic approaches: Transcranial electrical stimulation

Emerging evidence suggests that transcranial electrical stimulation (TES) may have antidepressant effects by modulating neuroinflammatory responses. In vivo imaging studies of rodent models have shown that TES activates both astrocytes and microglia (Monai et al., 2016; Mishima et al., 2019; Gellner et al., 2021), potentially restoring synaptic function and promoting neuroprotection. Transcranial direct current stimulation has been found to modulate inflammatory cytokine production via microglial pathways (Tan et al., 2023), supporting the hypothesis that electrical stimulation exerts its therapeutic effects through glial activation.

Given these findings, TES represents a promising non-invasive intervention for depression, particularly in cases where synaptic dysfunction and neuroinflammation play a prominent role in disease progression.

The interplay between inflammation, synaptic plasticity, and neuroendocrine dysfunction underlies depression pathophysiology. Chronic stress-induced HPA axis overactivation and inflammatory cytokine release lead to synaptic dysfunction, neurodegeneration, and prolonged depressive symptoms.

Future antidepressant strategies may need to target inflammation-mediated synaptic deficits. Promising approaches include: anti-inflammatory therapies (e.g., IL-1RA and TNF-α inhibitors), neuroprotective agents that enhance synaptic plasticity, and TES as a neuromodulatory treatment.

By addressing both neuroimmune and neuroplastic mechanisms, novel interventions may improve treatment efficacy and patient outcomes in inflammation-driven depressive disorders.

## Oxidative Stress and Its Role in Depression

### Mitochondrial dysfunction and oxidative stress in neuroinflammation

Mitochondria play a crucial role in both cellular energy metabolism and immune regulation. They integrate metabolic and neuroendocrine signals, modulate inflammatory responses, and regulate the cell cycle. Mitochondrial dysfunction has been implicated in numerous neuropsychiatric disorders, including depression, due to its role in inflammation and oxidative stress regulation (Noguchi and Kasahara, 2018; Filiou and Sandi, 2019; Kim et al., 2019).

The bacterial endosymbiosis theory suggests that mitochondria evolved from bacteria, and as a result, circulating cell-free mitochondrial DNA can act as damage-associated molecular patterns, triggering innate immune activation via TLRs (Xian et al., 2022). Dysfunctional mitochondria further exacerbate inflammation by promoting cytokine release, as demonstrated in a study where LPS-induced IL-6 production correlated with mitochondrial respiratory chain activity in human leukocytes (Huang et al., 2023). This finding supports the idea that early-life stress and immune dysfunction are linked via mitochondrial pathways.

Depression-related psychological stress increases cellular oxygen consumption in key brain regions, leading to the overproduction of ROS during mitochondrial oxidative phosphorylation. ROS can cause lipid peroxidation of neuronal membranes, accumulation of peroxides within neurons, reduced antioxidant defenses, and neuroinflammation.

Notably, ROS upregulates the NLRP3 inflammasome, a key mediator of neuroinflammation. Clinical studies indicate that mitochondrial ROS levels are significantly elevated in peripheral monocytes from depressed patients, correlating with NLRP3 inflammasome activation (Liu et al., 2025).

Experimental studies have confirmed that ROS generated from mitochondrial dysfunction activate inflammasomes via caspase-1. Oxidized mitochondrial DNA released into the cytoplasm serves as a pro-inflammatory signal, linking mitochondrial dysfunction to caspase-1 activation (Yu et al., 2025).

Further research has shown that ROS-TXNIP (thioredoxin-interacting protein)-NLRP3 signaling is upregulated in the hippocampus of rodents subjected to CUMS and acute LPS-induced depression models (Song et al., 2018; Dang et al., 2019). Astrocytes from uncoupling protein 2 knockout mice, which lack mitochondrial ROS regulation, exhibited even greater NLRP3 inflammasome activation following LPS stimulation (Du et al., 2016).

The inflammatory response culminates in gasdermin D-mediated pyroptosis, a form of inflammatory cell death. Activated caspase-1 cleaves gasdermin D, leading to microglial swelling and lysis, releasing pro-inflammatory cytokines IL-1β and IL-18 into the brain and sustaining neuroinflammation in depressed patients (He et al., 2015).

These findings underscore the critical role of ROS in inflammatory activation and suggest that mitochondrial dysfunction contributes to both oxidative stress and neuroinflammation, exacerbating depression-related pathology.

### Inflammation as a driver of oxidative stress

Inflammation and oxidative stress are interconnected pathways in depression pathology. Increased inflammation is associated with mitochondrial membrane depolarization, mitochondrial DNA oxidation, and elevated central and peripheral ROS production.

Depressed patients exhibit altered inflammatory markers and heightened oxidative stress responses (Samaryn et al., 2023). Rodent models of maternal separation stress have demonstrated that early-life adversity induces mitochondrial dysfunction, leading to increased ROS production, reduced adenosine triphosphate synthesis, and lower antioxidant capacity in the hippocampus (Fattahi Masrour et al., 2018; González-Pardo et al., 2020; Gumpp et al., 2020; Karan et al., 2020).

Interestingly, these mitochondrial abnormalities partially reversed following oxytocin treatment, suggesting that anti-inflammatory interventions may mitigate early-life stress-induced mitochondrial dysfunction (Matsushita et al., 2019).

A study by Gumpp et al. found that individuals with a history of childhood maltreatment exhibited higher energy demands and increased spontaneous inflammatory cytokine release (Gumpp et al., 2020). This suggests that early-life stress may drive mitochondrial dysfunction through immune system dysregulation, ultimately impacting central nervous system function.

### Mechanisms linking inflammation to oxidative stress

**Figure 5:**
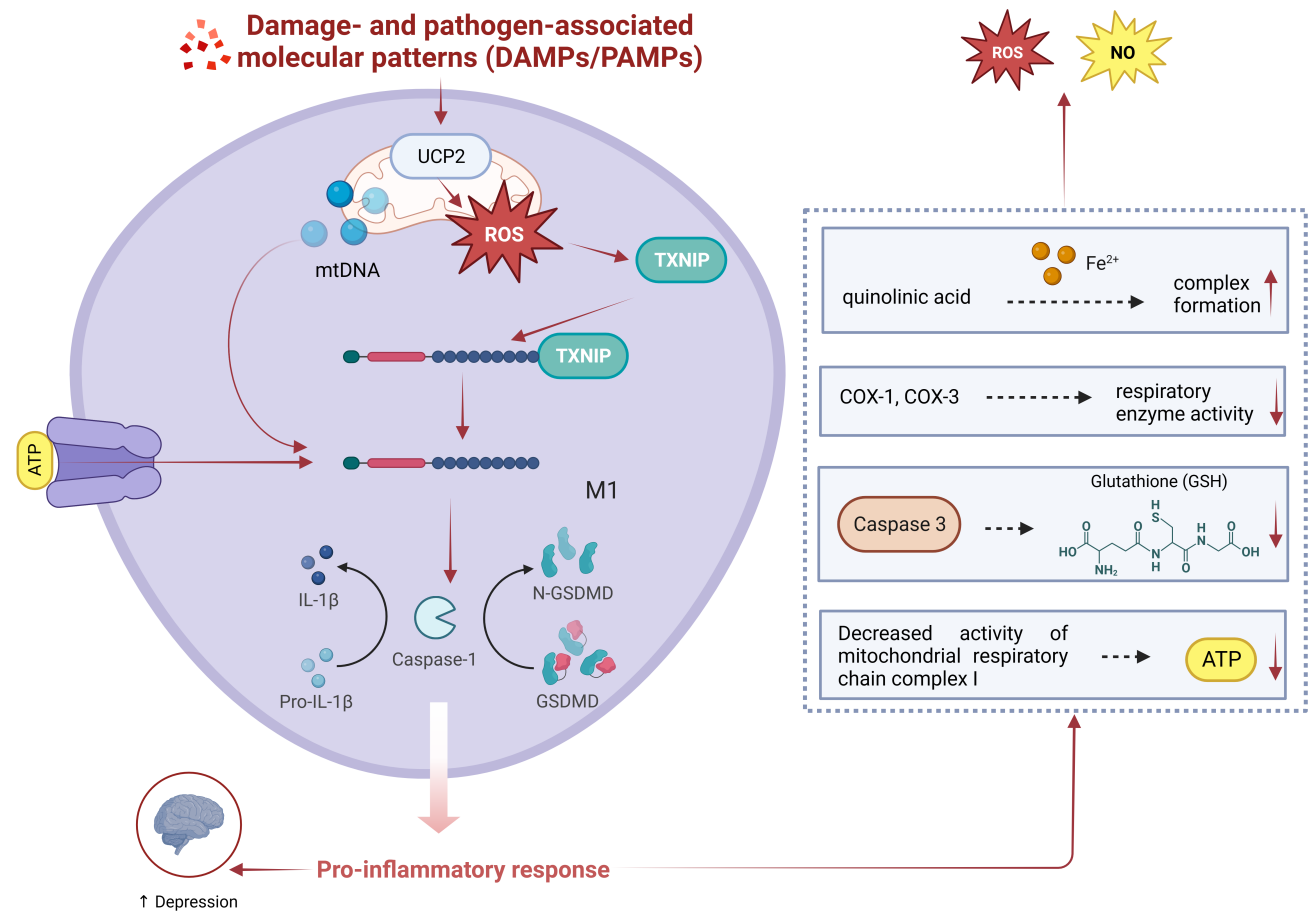
Relationship between mitochondrial disorders, oxidative stress, and inflammation. Pro-oxidant agents DAMPs and PAMPs induce elevated levels of ROS generation, which, in turn, triggers Toll-like receptors, and activates the innate immune system. However, when inflammation and cell-mediated immune activation occur, deleterious effects occur, such as membrane depolarization and damage to proteins and DNA/RNA, leading to mitochondrial and cellular dysfunction and ultimately central nervous system function. Elevated concentrations of ROS enhance neuroinflammation-related pathways and neuroinflammation. All these features form the pathophysiological basis of depression, which is crucial in the development and progression of the disease. Created with BioRender.com. ATP: Adenosine triphosphate; COX-1: cyclooxygenase-1; COX-3: cyclooxygenase-3; DAMPs: damage associated molecular patterns; GSDMD: gasdermin D; GSH: glutathione; IL-1β: interleukin-1 beta; mtRNA: mitochondrial ribosomal ribonucleic acid; NO: nitric oxide; PAMPs: pathogen-associated molecular patterns; Pro-IL-1β: interleukin-1β precursors; ROS: reactive oxygen species; TXNIP: thioredoxin interacting protein; UCP2: uncoupling protein 2.

Inflammation disrupts the balance between ROS production and metabolism, leading to oxidative stress via multiple pathways (**Figure 5**). Pro-inflammatory cytokines (e.g., TNF-α and IL-1β) inhibit mitochondrial respiratory chain complex I, reducing adenosine triphosphate synthesis and mitochondrial membrane potential. This impairs ROS metabolism and clearance, leading to an accumulation of oxidative species that contribute to oxidative stress. Some pro-inflammatory cytokines bind directly to apoptosis receptors, activating caspase-8 and caspase-3, which leads to glutathione depletion and further ROS accumulation (Wu et al., 2024). Inflammatory states increase metabolic demand, driving elevated mitochondrial activity and ROS overproduction. Excess nitric oxide and peroxynitrite (ONOO⁻) react with and damage lipids, proteins, and nucleic acids, contributing to mitochondrial and cellular dysfunction (Anderson et al., 2014; Zhu et al., 2025).

This chronic cycle of inflammation-induced oxidative stress is a key mechanism in depression-associated neurodegeneration.

### Implications for depression treatment

Given the interplay between oxidative stress, mitochondrial dysfunction, and neuroinflammation, targeting mitochondrial function and redox balance could offer novel therapeutic approaches for depression. Antioxidants and mitochondrial protectors (e.g., N-acetylcysteine, coenzyme Q10, and alpha-lipoic acid) could help reduce oxidative stress and inflammation-driven neuronal damage. IL-1β and TNF-α inhibitors might restore mitochondrial function and prevent oxidative stress-mediated depression. Pharmacological modulation of NLRP3 inflammasome activity may interrupt the inflammatory cascade and reduce depression severity.

These insights suggest that oxidative stress modulation could complement traditional antidepressants, offering new strategies for treating inflammation-driven depression.

## Gut Flora and Its Role in Depression

Emerging evidence suggests that gut microbiota play a crucial role in modulating depression, largely through their influence on inflammation, neurotransmitter production, and immune function. Studies in animal models have demonstrated that microbial changes in individuals exhibiting depressive-like behavior can be reversed through probiotic supplementation or fecal microbiota transplantation (Westfall and Pasinetti, 2019). These antidepressant effects of microbial transplantation have been linked to a reduction in inflammation (Cai et al., 2022).

In depressed patients, gut dysbiosis — an imbalance in gut microbial composition — has been associated with reduced production of anti-inflammatory metabolites, such as short-chain fatty acids (SCFAs). SCFAs help maintain intestinal barrier integrity and regulate immune responses; their depletion may predispose individuals to systemic inflammation and increased susceptibility to inflammatory diseases.

Additionally, peripheral immune signals may be transmitted to the CNS, providing a potential mechanistic link between gut microbiota, neuroinflammation, and depression.

### Gut-brain axis: A critical pathway in depression

While research on the gut microbiota-inflammation-depression connection has increased in recent years, comprehensive reviews on how these systems interact at a mechanistic level remain limited. The gut microbiota plays a fundamental role in maintaining brain homeostasis, as alterations in gut bacterial composition can disrupt intestinal barrier integrity, alter neurotransmitter metabolism, and affect immune system signaling.

The gut–brain axis, a bidirectional communication system between the gut and CNS, relies on microbial metabolites, immune interactions, and neural pathways to regulate brain function. The gut microbiota influences the CNS via multiple pathways.

(1) Metabolite signaling: Some gut microbial metabolites (e.g., neurotransmitters, vitamins, SCFAs) enter the bloodstream and modulate central immune responses, affecting neuroinflammation and synaptic activity (Xu et al., 2022).

(2) Immune activation: Gut microbiota can directly activate immune cells in the circulatory system, which then migrate to the CNS and modulate physiological functions in the brain (Pellegrini et al., 2020).

(3) Neuroinflammatory pathways: Chronic gut dysbiosis increases systemic inflammation, which may disrupt neurotransmitter synthesis, receptor function, and neuronal integrity.

The interaction between gut microbiota and the immune system plays a pivotal role in transferring information through the gut-brain axis. Disruptions in this system contribute to neuroinflammation and depressive symptomatology.

### Microbial–gut–inflammasome–brain axis

Recent research proposes the concept of the “microbial–gut–inflammasome–brain axis,” emphasizing the bidirectional communication between gut microbiota, immune signaling, and brain function (Inserra et al., 2018).

Chronic psychological stress has been shown to activate the NLRP3 inflammasome, triggering the release of IL-6 and IL-18, which disrupt intestinal barrier function, alter gut microbial composition, and increase systemic inflammation.

Studies in animal models have confirmed that gut dysbiosis leads to NLRP3 inflammasome activation, which, in turn, disrupts CNS homeostasis and contributes to depressive behaviors (Inserra et al., 2018). Zhang et al. (2019) demonstrated that NLRP3 gene deletion in mice altered gut flora composition and mood-related behaviors, suggesting that gut microbiota can regulate brain function via the NLRP3 inflammasome pathway.

This research supports the microbial–gut–inflammasome-brain hypothesis, which posits that psychological distress induces gut microbiota changes, these changes activate NLRP3 inflammasome pathways and immune signaling, resulting in immune dysregulation that contributes to depression and comorbid disorders.

Given the significant role of gut microbiota in inflammatory pathways, regulating gut flora and their metabolites may offer a novel strategy to reduce inflammatory cytokine secretion and improve depressive symptoms.

### Gut permeability, systemic inflammation, and depression

A key function of the gut microbiota is maintaining intestinal homeostasis and barrier integrity. However, factors such as chronic stress, dietary habits, and obesity can increase intestinal permeability, allowing microbial translocation into the bloodstream, which triggers immune activation and systemic inflammation.

When gut permeability is compromised, gut microorganisms and their metabolites enter circulation, eliciting a widespread immune response. This process increases the secretion of pro-inflammatory cytokines, such as IL-6 and IL-2.

A major driver of this inflammation is LPS, an endotoxin from intestinal bacteria. LPS activates TLR4 via CD14 clustering, stimulating monocytes to secrete pro-inflammatory cytokines, including IL-6 and TNF-α, leading to low-grade systemic inflammation (Yang et al., 2024).

Within the CNS, these inflammatory agents contribute to depressive symptoms by affecting neurotransmitter synthesis, neurotransmitter metabolism and reuptake, and neurotransmitter receptor expression.

### Zonulin, blood–brain barrier integrity, and depression

A critical factor linking gut permeability and neuroinflammation is zonulin, a protein that regulates endothelial and epithelial barrier permeability. Zonulin functions by disrupting tight junction proteins, such as zonula occludens-1 and occludin, increasing intestinal and BBB permeability (Berndt et al., 2019).

**Figure 6:**
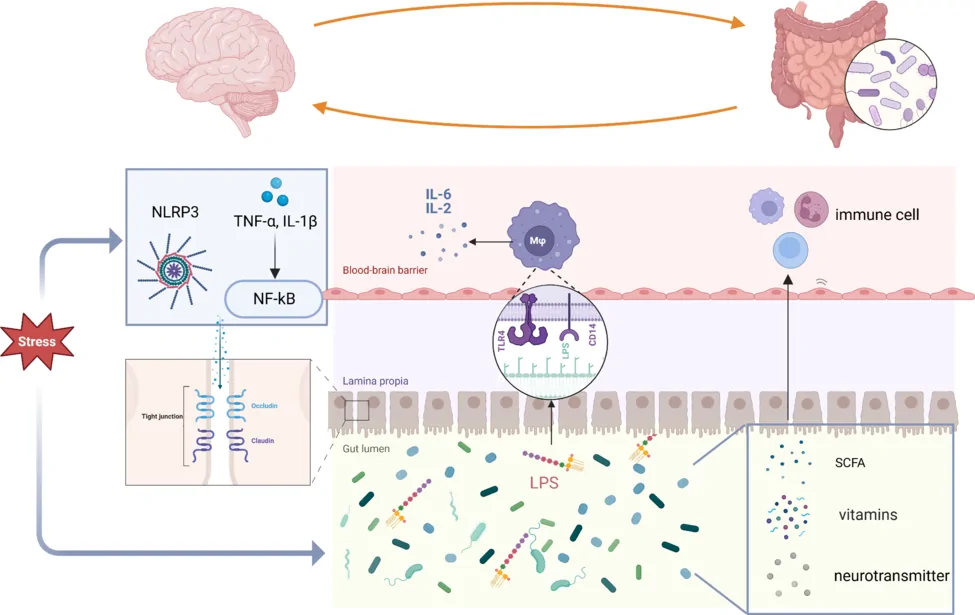
Relationship between gut flora and inflammation. Under stress, NLRP3 inflammatory vesicles are activated, and increased secretion of IL-1β and TNF-α cytokines leads to disruption of the integrity of the barrier. Consequently, the intestinal flora is also disrupted. Various microorganisms, as well as metabolites, may trigger activation of the immune system and inflammation within peripheral tissues. Various inflammatory factors, including TLR4, IL-2, and IL-6, enter the circulatory system through different pathways, disrupting the BBB, thus transmitting inflammatory signals to the center, where glial cell activation is promoted. Created with BioRender.com. CD14: Cluster of differentiation 14; IL-1β: interleukin-1β; IL-2: interleukin-2; IL-6: interleukin-6; LPS: lipopolysaccharide; NF-κB: nuclear factor kappa-B; NLRP3: NACHT-, LRR- and PYD-containing protein 3; SCFA: short chain fatty acids; TLR4: toll-like receptor 4; TNF-α: tumor necrosis factor-α.

Key findings include serum zonulin levels are significantly elevated in depressed patients compared to healthy controls. Pro-inflammatory cytokines (e.g., TNF-α, IL-1β) downregulate zonula occludens-1 expression via NF-κB activation, further increasing intestinal permeability. Cytokines such as TNF-α, interferon-γ, and IL-6 downregulate claudin-2 expression, further compromising gut mucosal integrity (**Figure 6**). This suggests that the gut may serve as both a source of systemic inflammation and a target of inflammatory responses.

A previous study shows that depressed patients exhibit elevated levels of circulating inflammatory markers, including interferon-γ, IL-1β, IL-6, and TNF-α (Lan et al., 2021). Since all of these cytokines influence intestinal permeability, gut microbiota dysfunction, and mucosal barrier impairment likely contribute to the chronic inflammation observed in depression.

### Therapeutic implications

The gut microbiota plays a fundamental role in neuroimmune regulation, with dysbiosis contributing to depression through increased intestinal permeability, systemic inflammation, and neuroinflammatory responses. Targeting the gut microbiome offers promising therapeutic avenues, including probiotics and prebiotics to restore microbial balance, boost anti-inflammatory metabolites such as SCFAs, and improve mood regulation. Fecal microbiota transplantation, effective in animal models, may help reestablish a healthy gut ecosystem, while dietary interventions, such as high-fiber, polyphenol-rich foods, fermented products, and omega-3 fatty acids, enhance microbial diversity and gut-brain communication. Additionally, zonulin inhibitors could restore gut and blood-brain barrier integrity, mitigating inflammation and depressive symptoms. By modulating gut flora, reducing inflammatory cascades, and repairing barrier function, these strategies may open new frontiers in treating depression and related mood disorders.

## Conclusions

MDD is a chronic, recurrent, and debilitating condition characterized by severe symptoms, a long duration, treatment resistance, and poor prognosis. Despite significant research, its complex pathophysiological mechanisms and lack of targeted treatment options continue to pose major clinical challenges.

This review explores MDD pathogenesis from an immunological perspective, emphasizing the growing body of evidence linking inflammation and depression. Inflammation is not only a precipitating factor for first-episode depression but also a major contributor to the chronicity of the disease. Chronic and repeated stress alters epigenetic modifications, leading to: elevated inflammatory cytokine levels, dysfunctional monoamine neurotransmission, neuroendocrine signaling imbalances, oxidative stress, gut microbiota disturbances, and alterations in synaptic plasticity. Ultimately, these changes result in structural and functional abnormalities in brain regions such as the hippocampus and medial prefrontal cortex, perpetuating depressive pathology.

However, the causal relationship between inflammation and depression remains an open question — does inflammation initiate depressive symptoms, or does depression drive inflammatory responses? This distinction is critical in determining whether inflammatory dysregulation is a universal characteristic of MDD or a defining feature of a specific depressive subtype (the inflammatory depression subgroup).

It is estimated that approximately 30% of individuals with depression exhibit elevated inflammatory markers and may benefit from anti-inflammatory treatment strategies (Lasselin, 2020). If inflammation is a core feature of a depressive subtype, identifying reliable inflammatory biomarkers could facilitate early and accurate diagnosis, personalized treatment, and targeted drug development.

### Role of inflammation in current antidepressant mechanisms

There is growing evidence that many clinically approved antidepressants exert anti-inflammatory effects on the CNS. For instance, venlafaxine, a serotonin-norepinephrine reuptake inhibitor, has been shown to inhibit neuroinflammatory responses by reducing microglial and astrocyte activation (Zhang et al., 2019b). Selective serotonin reuptake inhibitors exhibit moderate immunomodulatory effects, improving mood by modulating immune system activity (Wang et al., 2019). Minocycline, a second-generation tetracycline with anti-inflammatory properties, has demonstrated strong antidepressant effects. Research shows that interferon-γ activates microglia, impairing neurogenesis in the hippocampus and leading to depressive behaviors. However, minocycline administration inhibits microglial activation and neuroinflammation, effectively reversing depressive-like behaviors and cognitive deficits (Zhang et al., 2020a). Additionally, studies have confirmed that chronic stress-induced depression is mediated by neuroinflammation, which can be significantly attenuated by minocycline treatment (Kohler et al., 2016; Zhang et al., 2019a). This growing body of evidence supports the idea that suppressing neuroinflammation is a key mechanism through which many antidepressants exert their therapeutic effects.

### Future directions: Refining depression subtypes for precision medicine

Future research should focus on: (1) Refining depression subtypes: Developing biomarker-based classifications to distinguish inflammation-driven depression from other subtypes. Establishing more precise, biologically based diagnostic criteria for MDD. (2) Targeting neuroinflammation for antidepressant drug development: Investigating novel anti-inflammatory compounds as potential antidepressants. Exploring cytokine inhibitors, inflammasome blockers, and neuroimmune modulators. (3) Personalized treatment approaches: Identifying which patients will benefit most from anti-inflammatory treatments. Integrating immune-modulating strategies into existing antidepressant therapies. By establishing a more nuanced understanding of the depression’s inflammatory mechanisms underlying depression, we can develop more targeted and effective treatments, ultimately improving clinical outcomes for patients with MDD.

This review integrates current knowledge on the cellular and molecular underpinnings of MDD, with a focus on inflammation-induced impairment of neural regeneration. While preclinical data are promising, major challenges remain in translating these findings to clinical practice, particularly due to species differences, immune heterogeneity, and incomplete mechanistic understanding. Addressing these barriers requires interdisciplinary approaches combining neurobiology, immunology, and computational modeling. Targeting inflammatory cascades represents a promising frontier for novel antidepressants.

## Data Availability

*Not applicable.*
